# Occurrence of plant hormones in composts made from organic fraction of agri-food industry waste

**DOI:** 10.1038/s41598-024-57524-x

**Published:** 2024-03-21

**Authors:** Aneta Sienkiewicz, Małgorzata Krasowska, Małgorzata Kowczyk-Sadowy, Sławomir Obidziński, Alicja Piotrowska-Niczyporuk, Andrzej Bajguz

**Affiliations:** 1 Department of Agri-Food Engineering and Environmental Management, Faculty of Civil Engineering and Environmental Sciences, Bialystok University of Technology, Wiejska 45E, 15-351 Bialystok, Poland; 2 Department of Biology and Plant Ecology, Faculty of Biology, University of Bialystok, Ciolkowskiego 1J, 15-245 Bialystok, Poland

**Keywords:** Ecology, Environmental sciences

## Abstract

Utilizing the organic fraction of agri-food industry waste for fertilization represents one approach to waste management, with composting emerging as a popular method. Composts derived from this waste may contain plant hormones alongside primary macronutrients. This study aimed to evaluate the content of plant hormones in composts crafted from the organic fraction of agri-food industry waste. The presence of these substances was ascertained using liquid chromatography–mass spectrometry (LC-MS) analysis, applied to extracted samples from three composts produced in a bioreactor and three obtained from companies. The results indicate the presence of 35 compounds, which belong to six types of plant hormones: auxins, cytokinins, gibberellins, brassinosteroids, abscisic acid, and salicylic acid, in composts for the first time. The highest amount of plant hormones was noted in buckwheat husk and biohumus extract (35 compounds), and the lowest in hemp chaff and apple pomace (14 compounds). Brassinosteroids (e.g., brassinolide, 28-homobrassinolide, 24-epicastasterone, 24-epibrassinolide, and 28-norbrassinolide) and auxins (e.g., indolilo-3-acetic acid) are dominant. The highest concentration of total phytohormones was reported in biohumus extract (2026.42 ng g^−1^ dry weight), and the lowest in organic compost (0.18 ng g^−1^ dry weight).

## Introduction

The management of organic waste and residues from the agri-food industry leads to a reduction in problems related to waste management and environmental pollution^1^. Additionally, the appropriate processing of these materials aligns with the fundamentals of the circular economy (CE). The agri-food industry generates substantial amounts of organic waste and by-products, presenting a severe challenge for many food producers^2^. One of the most effective ways to manage organic waste is to utilize it for agricultural purposes, primarily fertilization, with composting being one of the methods. Composting involves the biological decomposition of selectively collected organic waste under controlled conditions by micro- and macroorganisms. Compost, a natural fertilizer, enhances soil structure, water retention capacity, and air–water relations^3^. Furthermore, compost is a multi-ingredient fertilizer, supplying nutrients vital for plant growth and offering beneficial microorganisms that positively influence the condition of the roots and overall plant health^4^. Employing compost derived from organic waste can also mitigate the adverse impact of soil salinity on plant growth and development^5^ and inhibit the uptake of heavy metals from soil^6^. Bożym and Rajmund^7^ reported that the issue of heavy metal pollution is primarily related to compost created from municipal waste and sewage sludge used as fertilizer. Therefore, compost is increasingly used in agriculture, significantly reducing the need for synthetic fertilizers. Researchers have explored the use of composts made from various organic waste from industries such as bakeries^8^, white wine production^9^, food processing^10^, brewing^11^, and herbal pharmaceuticals^12^. It is crucial to note that composts, made from an organic fraction of waste, can contain active plant growth substances that influence plant growth and development^13^, in addition to primary macronutrients, i.e., nitrogen, phosphorus, and potassium. Phytohormones, considered natural plant growth stimulators, are increasingly utilized in agriculture, horticulture, and forestry. They influence numerous physiological processes in plants include auxins (AXs), cytokinins (CKs), gibberellins (GAs), brassinosteroids (BRs), abscisic acid (ABA), and salicylic acid (SA). The roles of these plant hormones, particularly in plant growth and developmental processes, have been presented in numerous publications. AXs are crucial as they are involved in cell division, cell elongation, root formation, and the differentiation of cellular tissues. CKs influence cell division, thereby affecting plant growth, and also stimulate lateral buds and induce flowering, fruiting, and seed set. GAs promote germination, interrupt plant dormancy, and stimulate cell division. BRs regulate root and shoot growth, vascular differentiation, fertility, flowering, and seed germination. ABA plays a pivotal role in seed development, germination, vegetative growth, and the modulation of root architecture. SA is vital for DNA damage/repair, fruit yield, and seed germination^14–21^. Additionally, these phytohormones have a positive effect on organisms (human and animals) because they show antitumor, antidepressant, antioxidant and anti-stress activities^22^.

The content of plant hormones in composts derived from agri-food industry waste is seldom studied; hence, this aspect is pivotal since composts containing these growth substances can substitute synthetic fertilizers. Consequently, the present study focuses on evaluating the content of plant hormones, such as: AXs, CKs, GAs, BRs, ABA, and SA, in composts made from the organic fraction of agri-food industry waste using liquid chromatography–mass spectrometry (LC–MS) analysis. Notably, this study reports on a large number of plant hormones (35 compounds) in composts for the first time. Determining the content of phytohormones should be helpful in horticulture and agriculture for utilizing compost as organic fertilizers and agents that improve soil properties, as well as natural plant growth stimulants.

## Results and discussion

The research conducted revealed the presence of the following groups of phytohormones: auxins (AXs), brassinosteroids (BRs), cytokinins (CKs), gibberellins (GAs), abscisic acid (ABA), and salicylic acid (SA). Table 1 displays the content of phytohormones in composts. The average phytohormone content ranged from 0.13 to 2039.18 ng g^−1^ dry weight (dw). Brassinosteroids and AXs were the most prevalent, while GA_3_ was the least abundant.

**Table 1:** Average content (ng g^−1^ dw) and standard deviation (SD) of the studied groups of phytohormones in all tested composts.

Group of phytohormones	Average content	SD
Abscisic acid (ABA)	1.29	0.04
Auxins (AXs)	85.15	6.93
Brassinosteroids (BRs)	2039.18	154.64
Cytokinins (CKs)	15.74	1.28
GA_3_	0.13	0.01
Salicylic acid (SA)	4.33	0.38

**Figure 1:**
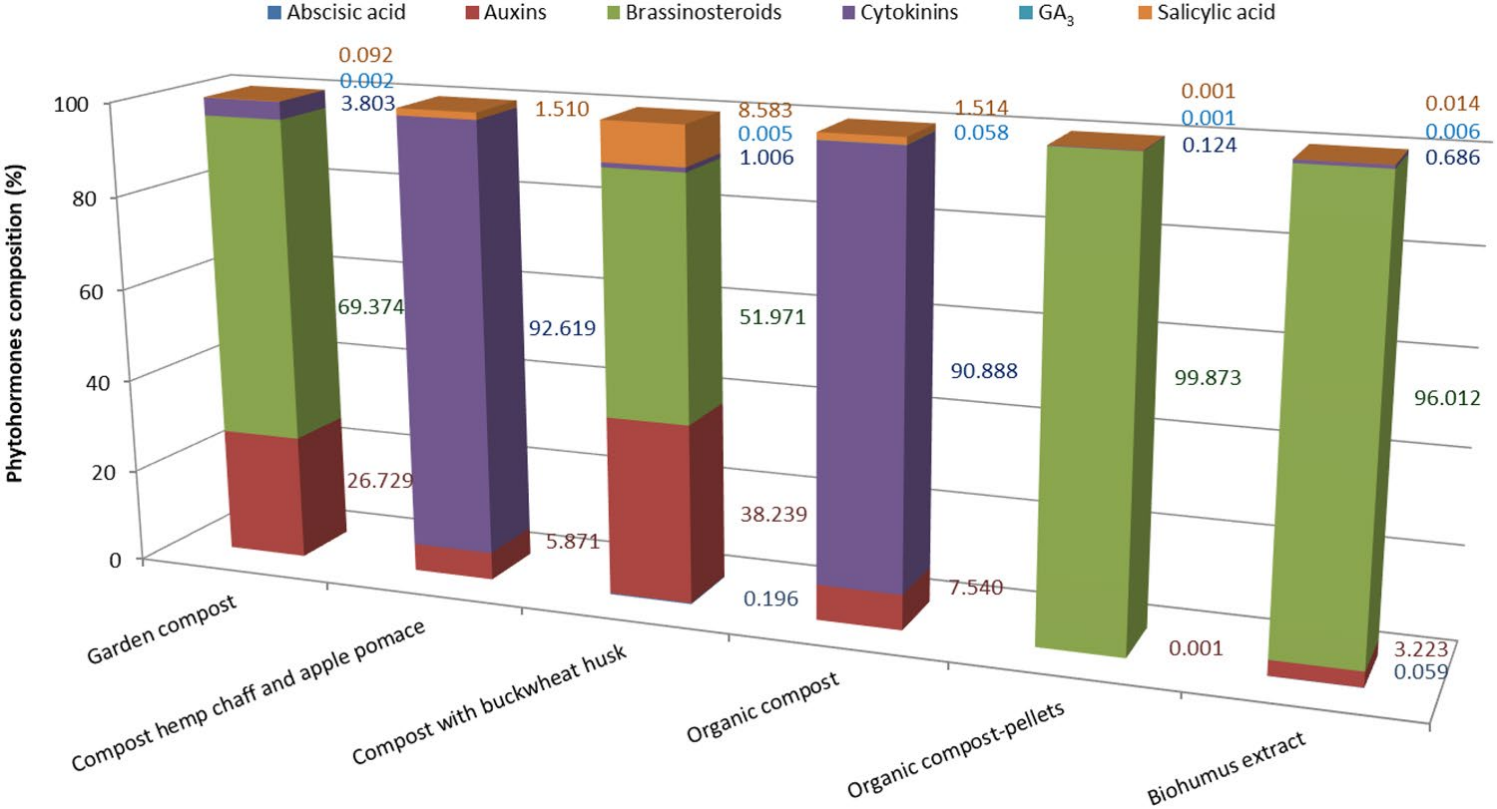
Phytohormones composition of analyzed composts from agri-food industry waste.

Auxins (AXs) and brassinosteroids (BRs) were identified as the prevailing types of phytohormones, as evidenced in Fig. 1.

The utmost concentration of BRs was found in organic compost-pellets, at 99.87%, and in biohumus extract, at 96.01%. Additionally, considerable quantities of brassinosteroids were present in garden compost and compost with buckwheat husk, registering 69.37% and 51.97%, respectively. Notably, compost hemp chaff and apple pomace, or purely organic sources did not exhibit detectable levels of BRs. Nevertheless, BRs have proven to be effective even in scant. They are recognized for encouraging cell division and elongation, influencing the growth of stems and roots, and playing a role in the initiation of floral structures and fruit development^21^. Moreover, Divi and Krishna^23^ highlighted the ability of BRs to safeguard plants against various stresses, both abiotic and biotic. Conversely, AXs exhibited their highest concentration in compost with buckwheat husk, at 38.24%, and their lowest in organic compost-pellets, at a mere 0.001%. Both AXs and BRs are integral to numerous physiological processes within plants^24,25^. Aremu et al.^26^ found a notably high AXs concentration, between 0.55 and 0.77 pmol mL^−1^, in vermicompost derived from garden waste. Additionally, Façanha et al.^27^ observed that substances from earthworm compost could spur lateral root development in maize plants.

Abscisic acid and GA_3_ show the lowest content in the evaluated waste, both less than 0.20% (Fig. 1). A peak ABA level of 0.20% was found in compost with buckwheat husk, while four types of compost, i.e., garden, hemp chaff and apple pomace, organic compost, and organic compost-pellets, exhibited no detectable levels of ABA. A study by Stirk et al.^28^ also reported low ABA concentration. Regarding GA_3_, the highest level was present in organic compost (0.06%), whereas the smallest was in compost from hemp chaff and apple pomace (< 0.001%). GAs, vital throughout the plant life cycle, promote stages like germination, hypocotyl elongation, and the development of organs, flowers, and seeds^29^. Over 90% CKs were found in compost hemp chaff and apple pomace, and organic sources. Salicylic acid levels peaked in compost with buckwheat husk at 8.58%, and plumbed to their lowest in organic compost-pellets at 0.001%.

An analysis using liquid chromatography–mass spectrometry (LC–MS) quantified the presence of up to 35 phytohormones in the compost samples. The various composts displayed a range of 14–35 distinct phytohormone types, i.e., compost with buckwheat husk and biohumus extract contained 35 compounds; compost hemp chaff and apple pomace, 14; organic compost, 15; organic compost-pellets, 21; and garden compost, 25 (Table 2).

**Table 2:** The content (ng g^−1^ dw) of phytohormones in composts from agri-food industry waste.

Phytohormone	Garden compost	Compost hemp chaff and apple pomace	Compost with buckwheat husk	Organic compost	Organic compost-pellets	Biohumus extract
ABA	ND	ND	0.09 ± 0.01^b^	ND	ND	1.20 ± 0.03^a^
IAA	1.86 ± 0.20^c^	0.03 ± 0.01^d^	15.66 ± 0.72^b^	< 0.01	ND	53.10 ± 5.03^a^
IBA	< 0.01	< 0.01	0.04 ± 0.01^b^	< 0.01	ND	3.63 ± 0.16^a^
IPA	< 0.01	< 0.01	2.20 ± 0.18^b^	< 0.01	< 0.01	7.67 ± 0.47^a^
PAA	< 0.01	< 0.01	0.02 ± 0.01^b^	< 0.01	ND	0.92 ± 0.16^a^
BL	4.69 ± 3.18^d^	ND	20.93 ± 1.56^c^	ND	62.27 ± 19.85^b^	688.75 ± 25.37^a^
HBL	< 0.01	ND	0.37 ± 0.01^b^	ND	0.10 ± 0.01^c^	327.17 ± 11.60^a^
epiBL	ND	ND	0.55 ± 0.08^b^	ND	0.16 ± 0.01^c^	271.78 ± 63.97^a^
norBL	< 0.01	ND	1.49 ± 0.01^b^	ND	0.25 ± 0.02^c^	239.76 ± 7.50^a^
epiCS	0.03 ± 0.01^d^	ND	0.91 ± 0.14^c^	ND	1.46 ± 0.22^b^	325.29 ± 14.87^a^
CT	< 0.01	ND	< 0.01	ND	< 0.01	< 0.01
6dTY	0.11 ± 0.01^c^	ND	0.09 ± 0.01^d^	ND	0.16 ± 0.01^b^	92.85 ± 6.22^a^
cZ	0.09 ± 0.01^c^	0.17 ± 0.01^b^	0.08 ± 0.01^c^	ND	ND	4.13 ± 0.49^a^
cZR	ND	ND	< 0.01	ND	ND	0.13 ± 0.01^a^
cZ9G	< 0.01	ND	0.03 ± 0.01^b^	ND	< 0.01	0.20 ± 0.01^a^
cZOGR	0.06 ± 0.01^c^	ND	0.06 ± 0.01^c^	ND	0.02 ± 0.01^b^	2.21 ± 0.06^a^
tZ	< 0.01	< 0.01	< 0.01	< 0.01	ND	0.10 ± 0.01^a^
tZR	ND	ND	< 0.01	ND	< 0.01	0.15 ± 0.01^a^
tZOG	ND	ND	0.03 ± 0.01	ND	< 0.01	1.21 ± 0.03
tZOGR	0.02 ± 0.01	0.05 ± 0.01	0.03 ± 0.01^b^	< 0.01	0.02 ± 0.01	0.74 ± 0.12^a^
tZ9G	ND	ND	< 0.01	< 0.01	< 0.01	0.34 ± 0.06^a^
DHZ	< 0.01	< 0.01	0.02 ± 0.01^b^	< 0.01	ND	0.49 ± 0.01^a^
DHZR	ND	ND	< 0.01	ND	ND	< 0.01
DHZOG	ND	ND	< 0.01	ND	< 0.01	< 0.01
DHZOGR	0.02 ± 0.01^d^	ND	0.02 ± 0.01^c^	0.07 ± 0.01^b^	0.02 ± 0.01^d^	0.75 ± 0.12^a^
DHZ7G	0.02 ± 0.01^c^	0.20 ± 0.01^a^	< 0.01	ND	< 0.01	0.12 ± 0.02^b^
DHZ9G	ND	ND	< 0.01	ND	< 0.01	0.13 ± 0.02^a^
IP	0.05 ± 0.01^d^	0.43 ± 0.02^b^	< 0.01	0.07 ± 0.01^c^	ND	0.93 ± 0.03^a^
IPR7G	ND	ND	0.09 ± 0.01^b^	ND	< 0.01	0.22 ± 0.01^a^
oT	< 0.01	ND	< 0.01	ND	ND	0.18 ± 0.01^a^
mT	< 0.01	< 0.01	< 0.01	< 0.01	ND	0.17 ± 0.01^a^
pT	< 0.01	< 0.01	< 0.01	< 0.01	ND	0.13 ± 0.01^a^
BA	< 0.01	< 0.01	< 0.01	< 0.01	ND	1.56 ± 0.14^a^
GA_3_	< 0.01	ND	< 0.01	< 0.01	< 0.01	0.13 ± 0.01^a^
SA	< 0.01	< 0.01	4.02 ± 0.37^a^	< 0.01	< 0.01	0.28 ± 0.01^b^
Total number of compounds	25	14	35	15	21	35

Data represent the mean (n = 4) ± standard deviation. Means with the same letters are not significantly different (*p* ≥ 0.05) according to Tukey’s post hoc test. ND, not detected.

**Figure 2:**
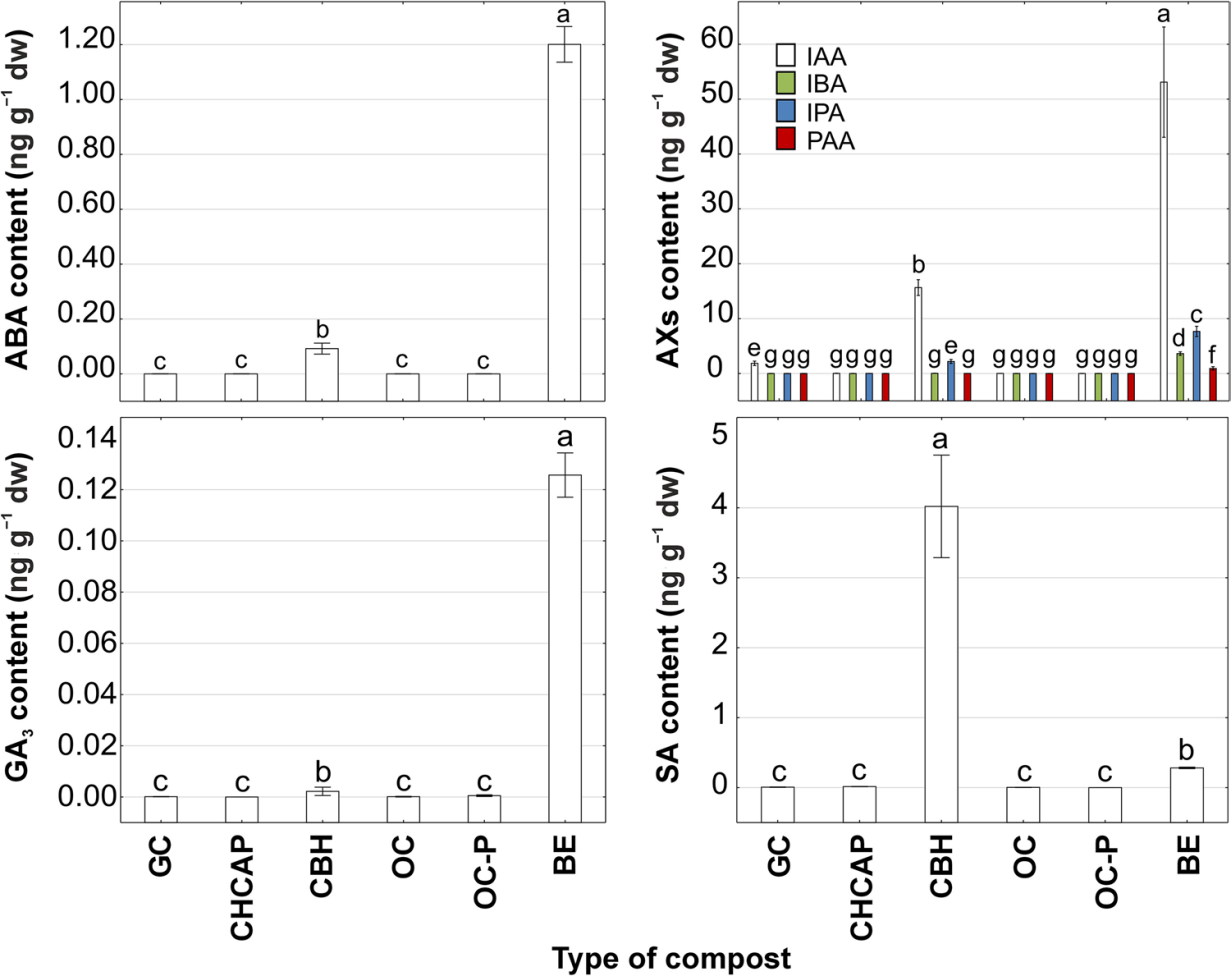
The content of abscisic acid (ABA), auxins (AXs), gibberellic acid (GA_3_) and salicylic acid (SA) in different type of compost. Data represent the mean (n = 4) ± standard deviation. Means with the same letters are not significantly different (*p* ≥ 0.05) according to Tukey’s post hoc test. Abbreviations of composts: GC, garden compost; CHCAP, compost hemp chaff and apple pomace; CBH, compost with buckwheat husk; OC, organic compost; OC-P, organic compost-pellets; BE, biohumus extract.

Biohumus extract exhibited the highest concentration of total phytohormones at 2026.42 ng g^−1^ dw, while organic compost had the lowest at 0.18 ng g^−1^ dw (Table 3). The compost with buckwheat husk showcased the highest content of SA (4.02 ng g^−1^ dw), whereas GA_3_ was the least concentrated hormones across all composts (Fig. 2).

**Table 3:** Sum of mean phytohormones content ± standard deviation (SD) (ng g^−1^ dw) in composts both with and without distinction for abscisic acid, auxins, brassinosteroids, cytokinins, GA_3_, and salicylic acid.

Composts (ng/g dw)	Abscisic acid	Auxins	Brassinosteroids	Cytokinins	GA_3_	Salicylic acid	∑ Phytohormones
Garden compost	ND	1.86 ± 0.20	4.83 ± 3.19	0.27 ± 0.04	< 0.01	< 0.01	6.96 ± 3.43
Compost hemp chaff and apple pomace	ND	0.05 ± 0.01	ND	0.87 ± 0.05	ND	< 0.01	0.93 ± 0.06
Compost with buckwheat husk	0.09 ± 0.01	17.91 ± 0.90	24.35 ± 1.80	0.47 ± 0.05	< 0.01	4.02 ± 0.37	46.85 ± 3.13
Organic compost	ND	< 0.01	ND	0.17 ± 0.02	< 0.01	< 0.01	0.18 ± 0.02
Organic compost-pellets	ND	< 0.01	64.40 ± 20.12	0.08 ± 0.03	< 0.01	< 0.01	64.48 ± 20.15
Biohumus extract	1.20 ± 0.03	65.31 ± 5.81	1945.60 ± 129.54	13.90 ± 1.16	0.13 ± 0.01	0.28 ± 0.01	2026.42 ± 136.56

ND, Not detected.

**Figure 3:**
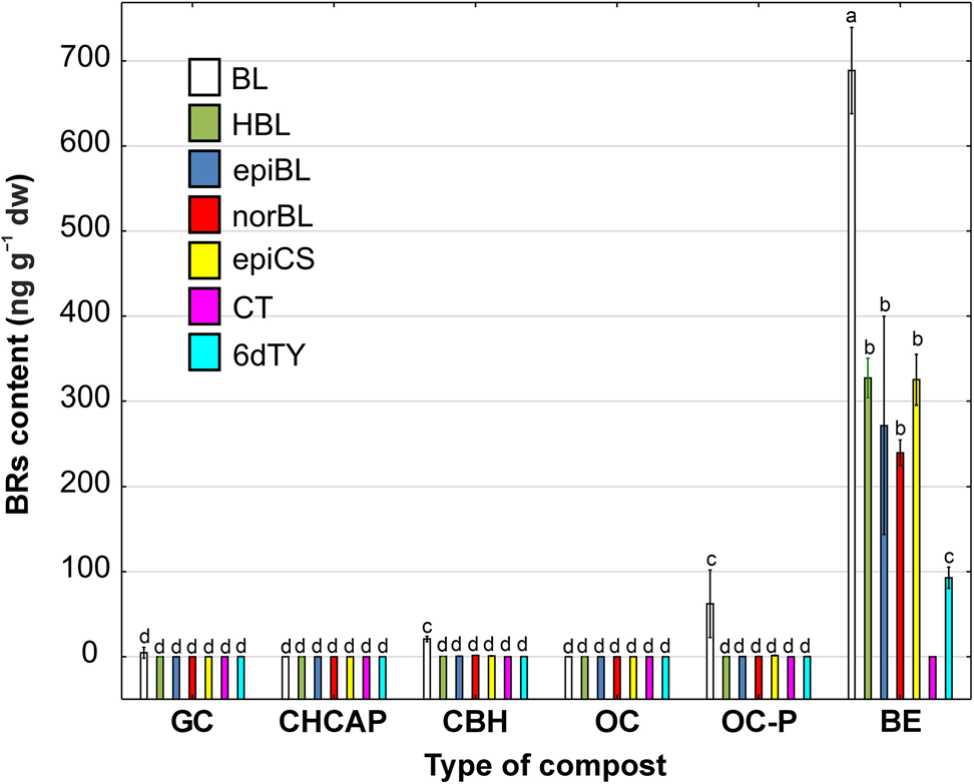
The content of brassinosteroids (BRs) in different type of compost. Data represent the mean (n = 4) ± standard deviation. Means with the same letters are not significantly different (*p* ≥ 0.05) according to Tukey’s post hoc test. Abbreviations of composts: GC, garden compost; CHCAP, compost hemp chaff and apple pomace; CBH, compost with buckwheat husk; OC, organic compost; OC-P, organic compost-pellets; BE, biohumus extract.

Every type of phytohormone was detectable in both compost with buckwheat husk and biohumus extract. Dominant phytohormones in composts derived from agri-food industry waste included IAA (Fig. 2), BL, HBL, epiCS, epiBL, and norBL (Fig. 3).

**Figure 4:**
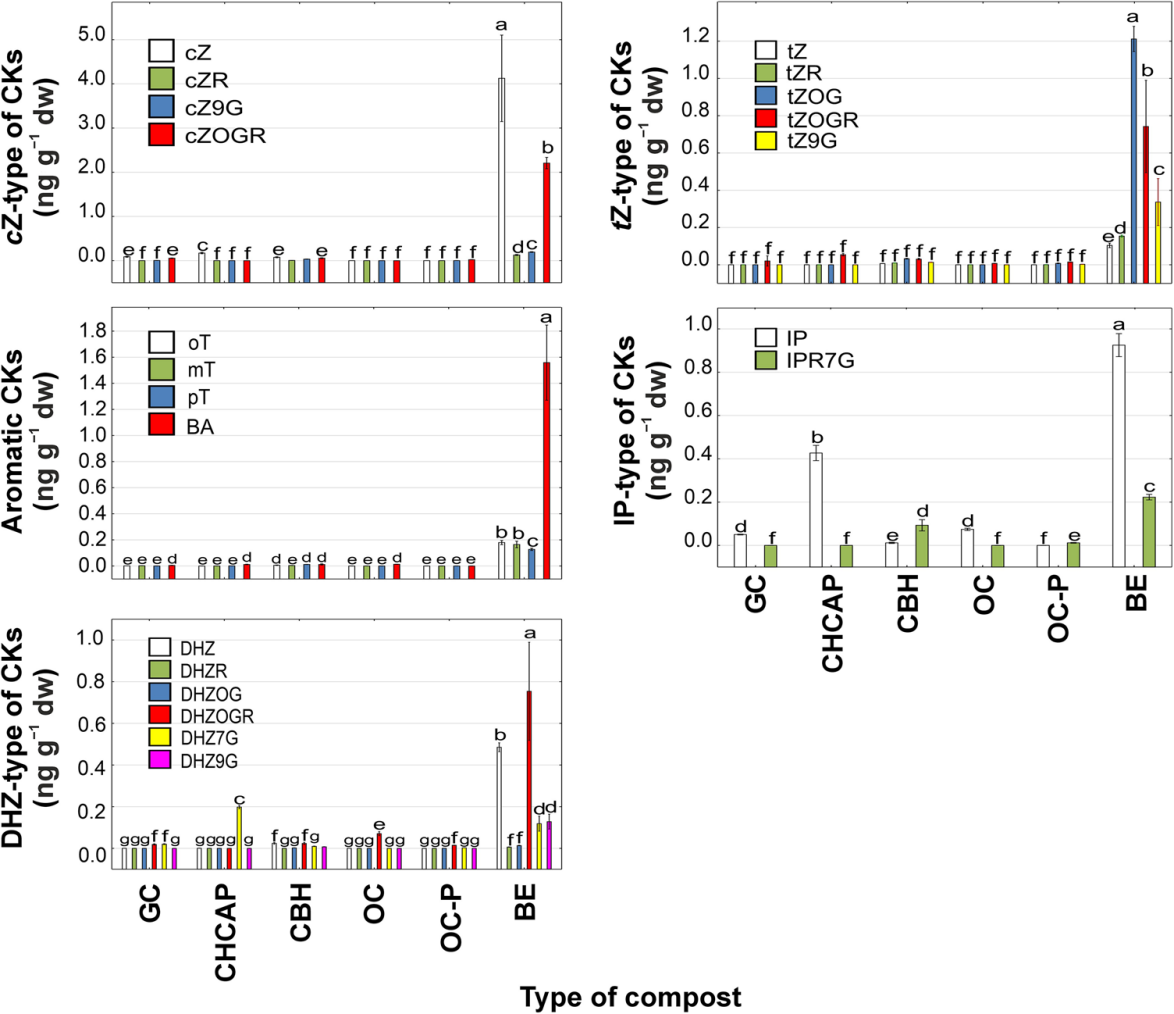
The content of cytokinins (CKs) in different type of compost. Data represent the mean (n = 4) ± standard deviation. Means with the same letters are not significantly different (*p* ≥ 0.05) according to Tukey’s post hoc test. Abbreviations of composts: GC, Garden compost; CHCAP, Compost hemp chaff and apple pomace; CBH, Compost with buckwheat husk; OC, Organic compost; OC-P, Organic compost-pellets; BE, Biohumus extract.

cZ-type of CKs were not detected in organic compost and aromatic CKs were not noted in organic compost-pellets (Table 4). Dominant type of CK group in composts was cZ-type (Fig. 4). Schäfer et al.^30^ summarized studies that investigate the role of this type of CK in regulating plant development and defense responses to pathogen and herbivore attack. It can be concluded that the diversity in phytohormone content across composts is influenced by the type of substrates utilized during the composting process^13,31^.

**Table 4:** Sum of mean content of cytokinins (CKs) types ± standard deviation (SD) (ngg^−1^ dw) in the tested composts.

Composts	Total CK content	cZ-type	tZ-type	DHZ-type	IP-type	Aromatic CK
Garden compost	0.27 ± 0.04	0.15 ± 0.01	0.02 ± 0.01	0.04 ± 0.01	0.05 ± 0.01	< 0.01
Compost hemp chaff and apple pomace	0.87 ± 0.05	0.17 ± 0.01	0.06 ± 0.01	0.20 ± 0.01	0.43 ± 0.02	< 0.01
Compost with buckwheat husk	0.47 ± 0.05	0.17 ± 0.01	0.09 ± 0.01	0.07 ± 0.01	0.11 ± 0.01	0.03 ± 0.01
Organic compost	0.17 ± 0.02	ND	< 0.01	0.07 ± 0.01	0.08 ± 0.01	< 0.01
Organic compost-pellets	0.08 ± 0.03	0.02 ± 0.01	0.03 ± 0.01	0.02 ± 0.01	< 0.01	ND
Biohumus extract	13.90 ± 1.16	6.66 ± 0.56	2.55 ± 0.23	1.51 ± 0.17	1.15 ± 0.03	2.03 ± 0.17

ND, not detected.

Our findings suggest a notably elevated content of plant hormones in the leachate derived from composted hemp chaff and grass. The overall content of the plant hormones analyzed spanned from 1456.32 to 4094.34 ng g^−1^ dw, averaging at 462.56 ng g^−1^ dw. This leachate demonstrated the highest concentrations of BRs (ranging from 661.66 to 3368.31 ng g^−1^ dw), AXs (324.75–336.73 ng g^−1^ dw), and SA (300.31–350.55 ng g^−1^ dw). Arthur et al.^32^ and Aremu et al.^25^ have noted that leachate from thoroughly decomposed compost contains CK-like substances, originating from the hydrolysis of CK glucosides by β-glucosidase, an enzyme produced by microbes. Subsequent research should, therefore, prioritize evaluating the content of plant hormones in leachates, specifically those derived from composts produced from the organic fraction of agri-food industry waste^33^.

**Figure 5:**
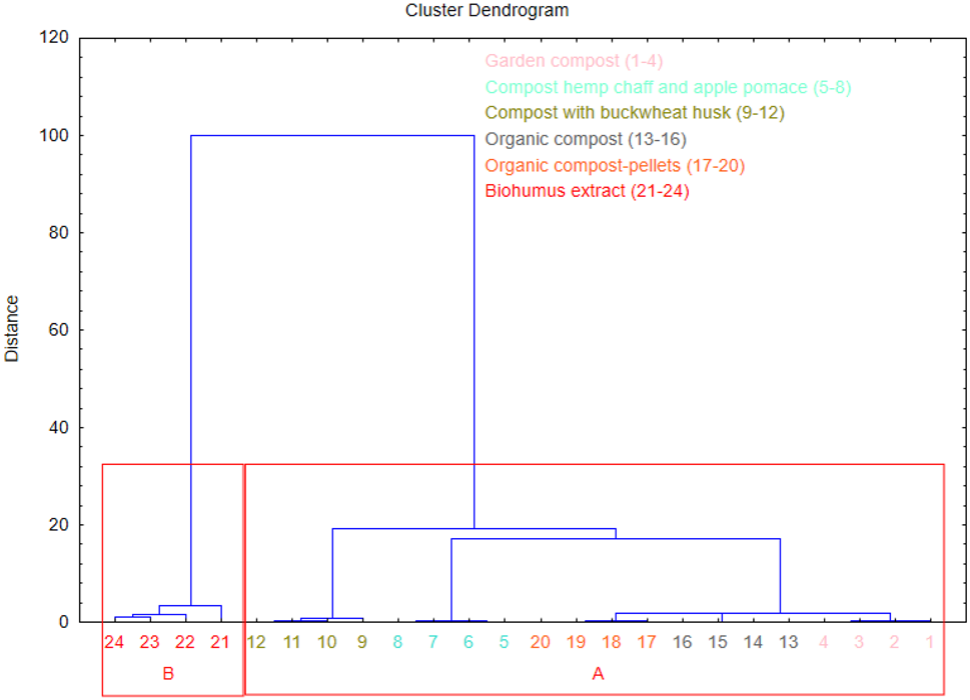
Dendrogram of the hierarchical cluster analysis of six types of composts. Final partition: Cluster A: garden compost, compost hemp chaff and apple pomace, compost with buckwheat husk, organic compost, organic compost-pellets, Cluster B: biohumus extract.

Hierarchical Cluster Analysis effectively delineated the assessed composts into two discrete clusters, denominated as A and B, based on the constituent phytohormone content (Fig. 5). Cluster A, encompassing garden compost, compost derived from hemp chaff and apple pomace, compost with an inclusion of buckwheat husk, organic compost, and analogous organic compost in a pelletized configuration, exhibits a phytohormone composition that is discernibly lower in concentrations of ABA, AXs, BRs, CKs, and GA_3_ relative to the mean of the group; notwithstanding, it is punctuated by the pinnacle of SA concentration within this cluster. Such a phytohormone profile implicates potential limitations of these compost variants for agricultural applications. Conversely, cluster B, exclusively represented by biohumus extract, albeit exhibiting diminished SA content, is distinctly characterized by superlative concentrations of ABA, AXs, BRs (i.e., BL, HBL, epiCS, epiBL, and norBL), CKs, and GA_3_, thereby positing it as a potentially preeminent stimulator of plant growth.

**Figure 6:**
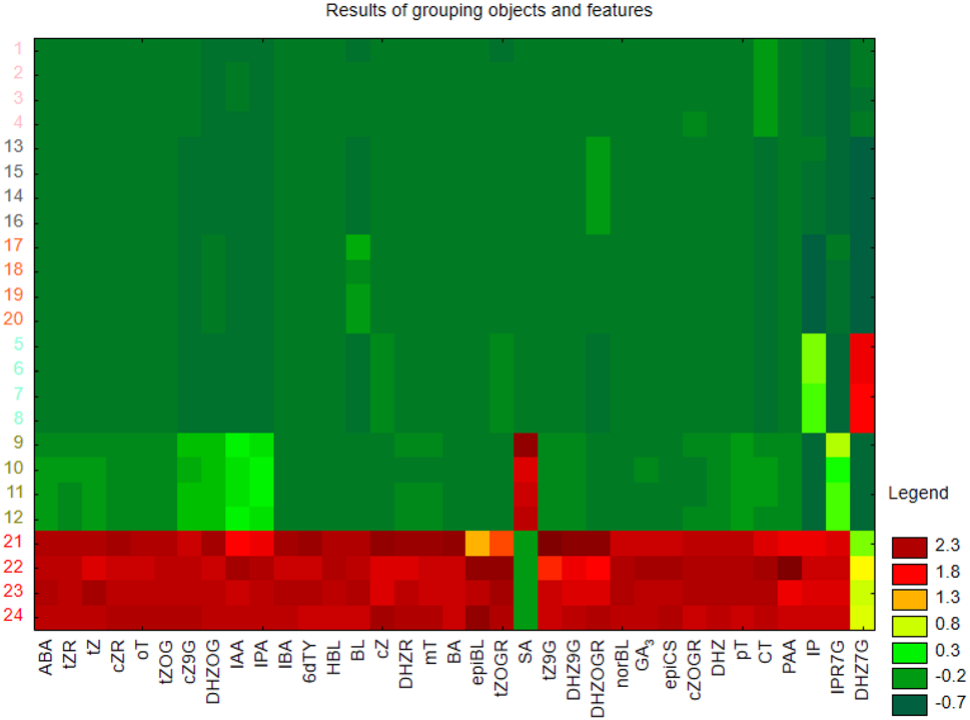
Graphical result of the simultaneous grouping of objects (tested composts, the numbers correspond to the composts from the dendrogram) and features (content of plant hormones).

The analysis of plant hormone content in the examined composts revealed two distinctly dissimilar groups of objects when objects and features were simultaneously grouped (Fig. 6). In the upper part of the map, an area was obtained where the garden compost, organic compost, organic compost in the form of pellets, compost from hemp chaff and apple pomace, and compost with buckwheat husk were grouped (green color). Conversely, in the lower part of the map, an area was designated where the biohumus extract was grouped (red color).

**Figure 7:**
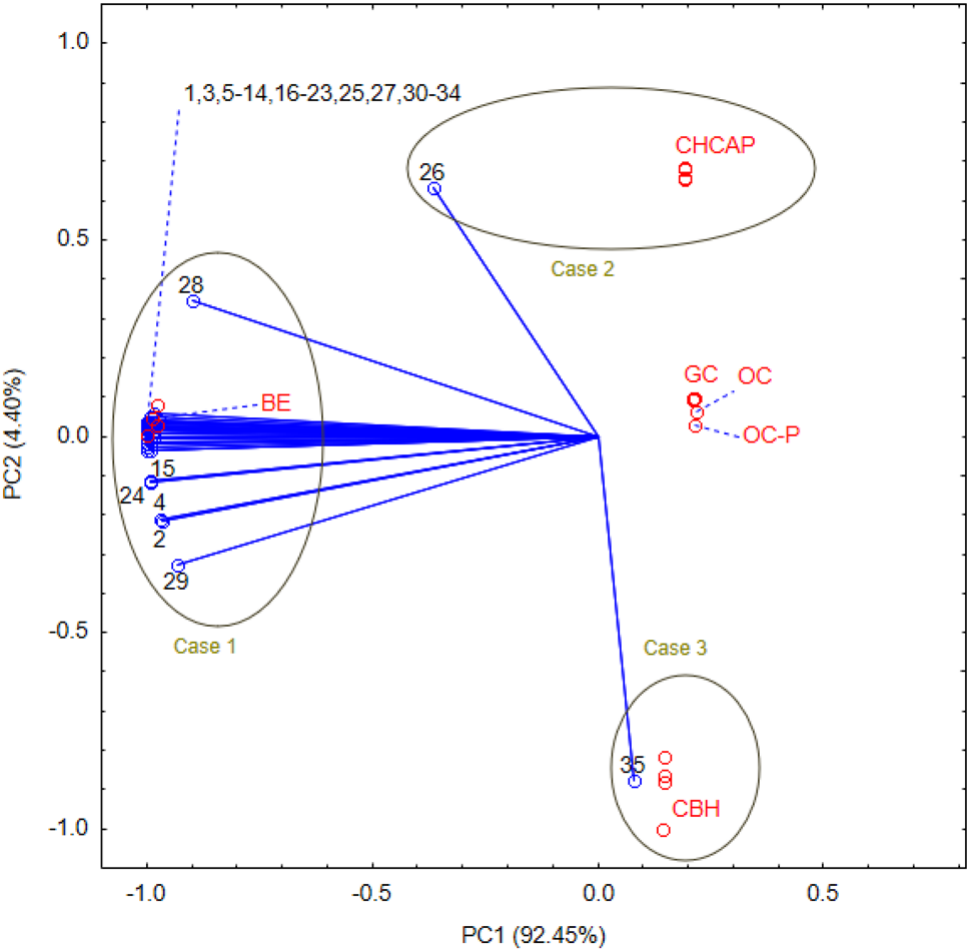
Biplot of plant hormones content for each repetition (n = 4) in six types of composts, showing the first two principal components (PC1 and PC2) of the PCA model that together explain 96.9% of the total variance, i.e., 92.45% and 4.40% for PC1 and PC2, respectively. Blue biplot vectors indicate the strength and direction of factor loading for all analyzed variables: 1, ABA; 2, IAA; 3, IBA; 4, IPA; 5, PAA; 6, BL; 7, HBL; 8, epiBL; 9, norBL; 10, epiCS; 11, CT; 12, 6dTY; 13, cZ; 14, cZR; 15, cZ9G; 16, cZOGR; 17, tZ; 18, tZR; 19, tZOG; 20, tZOGR; 21, tZ9G; 22, DHZ; 23, DHZR; 24, DHZOG; 25, DHZOGR; 26, DHZ7G; 27, DHZ9G; 28, IP; 29, IPR7G; 30, oT; 31, mT; 32, pT; 33, BA; 34, GA_3_; 35, SA. Composts are marked in red: BE, biohumus extract; CHCAP, compost hemp chaff and apple pomace; GC, garden compost; OC, organic compost; OC-P, organic compost-pellets; CBH, compost with buckwheat husk.

PCA analysis enabled the categorization of analyzed composts, maintaining a significant degree of explained variance. During this analysis, the variable count was condensed to two principal components (designated as PC1 and PC2), which intimates that the initial dataset of 35 plant hormones is notably correlated and therefore reducible (Fig. 7). All variables, with the exception of DHZ7G and SA, presented high negative loadings, ranging from − 0.9996 to − 0.8995, in association with the first component. Contrastingly, DHZ7G was associated with a high positive loading (0.6334), whereas SA exhibited a high negative loading (− 0.8768) with the second component. The values of factor loadings for most variables were proximate, resulting in a superimposition of points on a singular graph.

**Figure 8:**
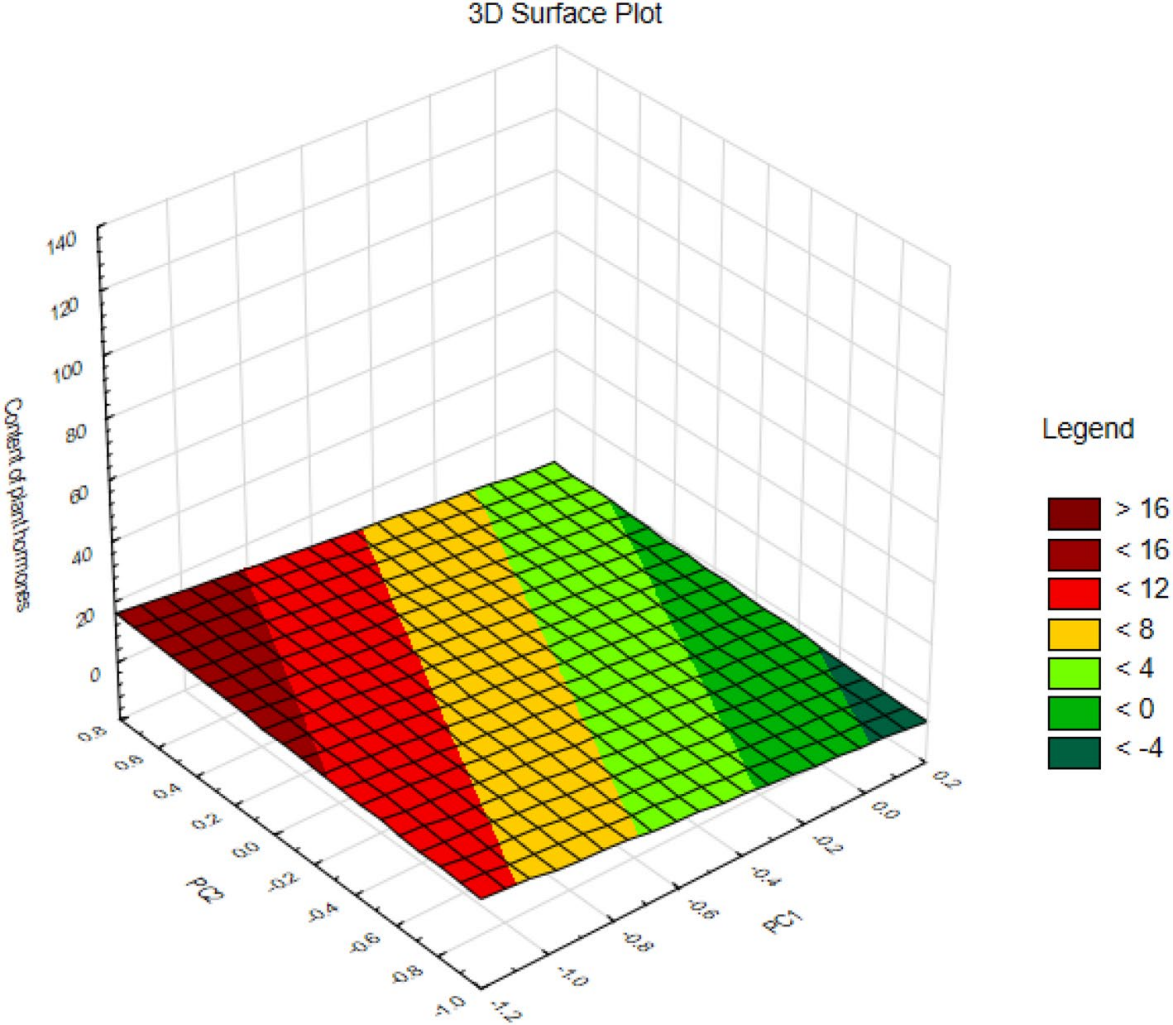
A 3D-surface plot showing the relationship among the content of plant hormones, PC1 and PC2.

Comparison of case positions on the graph, considering component forms and factor loadings, revealed distinct characteristics among them. Specifically, Case 1, exhibiting negative coordinate values on the first axis, was identified as having a higher content of plant hormones, excluding DHZ7G and SA, according to the relevant factor loadings. Conversely, Case 2, with a positive coordinate value for the second axis, was associated with a higher DHZ7G content, based on the factor loading with the second axis. In contrast, Case 3, showing a negative coordinate value on the second axis, was characterized by a higher SA content, based on its factor loading with the second axis (Fig. 7). The interplay between the content of plant hormones and the two principal components (PC1 and PC2) is visually represented in a three-dimensional surface plot (Fig. 8).

## Conclusions

The variation in plant hormone content within the tested organic composts was influenced by the choice of substrates utilized during the composting process. Brassinosteroids, identified with notable prevalence in the tested composts, could potentially expedite plant growth, as well as trigger the onset of flowering and fruit development. The compost comprising buckwheat husk and biohumus extract demonstrated the most significant phytohormone content variation and was established to contain all recognized groups of plant hormones. Characterized by the highest total phytohormone concentration, biohumus extract is posited as the optimal natural stimulant for plant growth. It has been ascertained that the pelleting process can modify phytohormone content, evidenced by an elevated concentration of brassinosteroids in compost post-pelletization relative to its pre-pelletization state.

## Materials and methods

### Characteristics of composts

**Figure 9:**
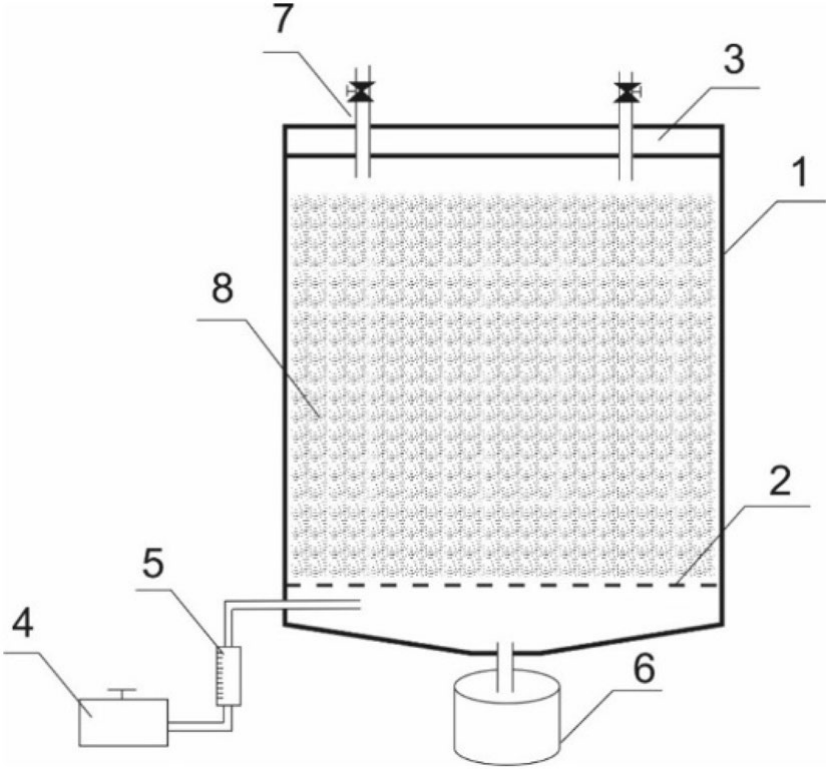
Diagram of a laboratory bioreactor: 1—enclosure, 2—perforated bottom, 3—sealed cover, 4—air pump, 5—flow meter, 6—leachate container, 7—air vents with air flow regulation, 8—composted biomass.

The composted wastes were collected from facilities located in the Podlasie Voivodeship (Poland), an area where the predominant sectors of the agri-food industry include milk, meat, fruit and vegetable processing plants. The composts were formulated using the organic fraction of agri-food industry waste and residues from fruit and vegetable processing, all within a laboratory bioreactor with engineered aeration (Fig. 9, Table 5). The study entailed crafting experimental compost mixtures through a two-stage composting process. Initially, the composting process involved a phase of notably intense decomposition of the organic fraction at temperatures ranging from 60 to 75 °C. Subsequently, an intense yet diminishing decomposition of the organic fraction occurred over time, leading to a gradual temperature decrease to between 30 and 40 °C. The bioreactor composting process spanned 14 days. In Table 5 the composition of tested composts obtained from companies was also presented.

**Table 5:** Composition of the tested composts.

Composts	Composition
Produced in a bioreactor	
Garden compost	Green waste (grass, leaves, branches)
Compost from agricultural and food processing residues (1)	Hemp chaff, apple pomace
Compost from agricultural and food processing residues (2)	Grass, buckwheat husk, fruit pomace
Obtained from companies	
Organic compost	Green waste
Organic compost-pellets	Green waste
Biohumus extract	Residues from agricultural and food processing

**Figure 10:**
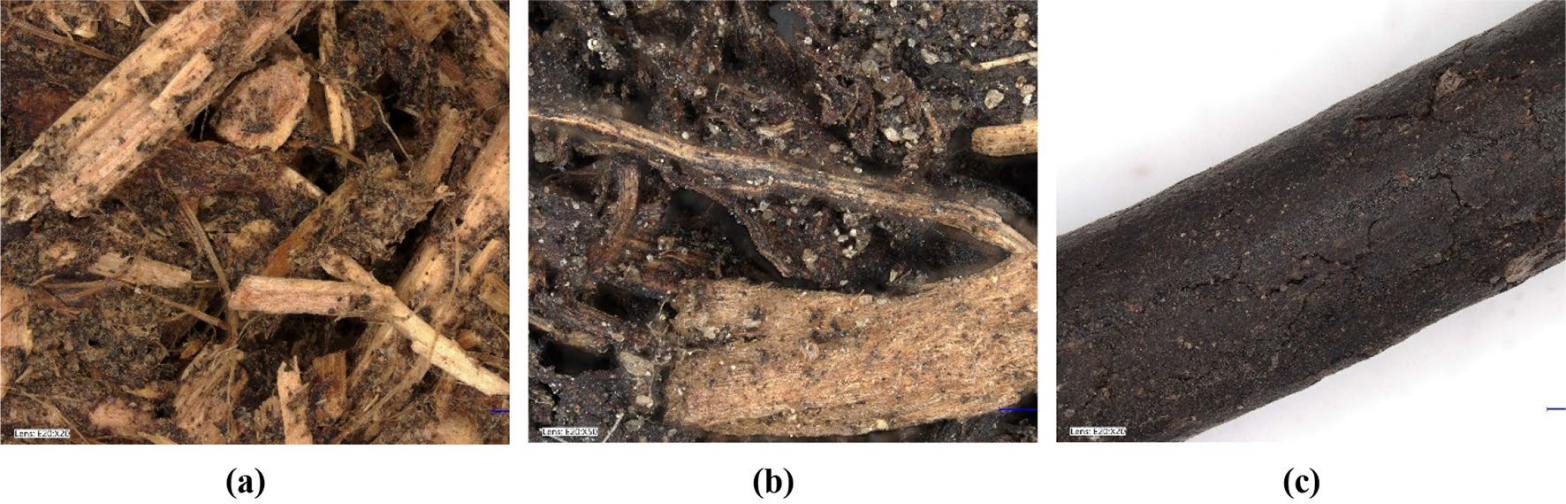
Microstructure image of (**a**) compost hemp chaff and apple pomace, (**b**) compost with buckwheat husk and (**c**) organic compost-pellets from Keyence Digital Microscope.

The compost blends were formulated considering the fundamental characteristics such as pH, dry matter content, organic matter, and elements like nitrogen, phosphorus, potassium, and carbon from the processing waste used. Details on the physical and chemical parameters of specific agri-food processing residues were previously documented^34^. The composting process yielded fully matured composts, distinguishable by their dark brown hue, consistent texture, and a distinctive fresh soil aroma (Fig. 10a,b). The organic solid compost was additionally subjected to a granulation process under laboratory conditions to evaluate the impact of thermal processing (Fig. 10c).

In the analyzed composts, nitrogen was determined by acid digestion using a catalyst, phosphorus was determined by mineralization to phosphorus (V) followed by a spectrophotometric method, and potassium was evaluated by digestion with an acid mixture, and subsequently determined by atomic spectrometry^35^. The values of the total solids (TS) content were determined using the standard PN-EN14346:2011^36^, while the values of the volatile solids (VS) content were determined using the standard PN-Z-15011-3:2001^35^. Carbon was determined throught catalytic oxidation. Table 6 shows the results from evaluating the fertilizing properties of the finalized composts, obtained under laboratory conditions.

**Table 6:** Selected properties of the tested composts.

Type of tested composts	pH	TS	VS	Carbon	Nitrogen	Phosphorus	Potassium
	–	(%)	(%)	(% dw)	(% dw)	(% dw)	(% dw)
Garden compost	6.8 ± 0.28	32 ± 1.21	61 ± 2.56	7.2 ± 0.67	1.8 ± 0.08	0.9 ± 0.04	0.4 ± 0.02
Compost from agricultural and food processing residues (1)	6.1 ± 0.22	34 ± 1.14	57 ± 3.06	6.9 ± 1.42	1.5 ± 0.06	0.9 ± 0.08	0.5 ± 0.04
Compost from agricultural and food processing residues (2)	6.7 ± 0.25	29 ± 1.32	58 ± 3.14	7.3 ± 0.89	1.9 ± 0.04	1.2 ± 0.11	0.8 ± 0.03
Organic compost	5.9 ± 0.23	33 ± 1.25	63 ± 3.77	6.4 ± 1.25	1.4 ± 0.10	0.4 ± 0.06	0.6 ± 0.04
Organic compost-pellets	6.4 ± 0.27	29 ± 1.47	64 ± 4.85	6.2 ± 0.76	1.2 ± 0.92	0.8 ± 0.03	0.4 ± 0.01
Biohumus extract	5.6 ± 0.24	18 ± 1.05	58 ± 5.77	7.4 ± 1.63	0.6 ± 0.05	0.1 ± 0.04	0.1 ± 0.03

### Chemicals

All chemicals used in phytohormone extraction and salicylic acid (SA) were purchased from Merck KGaA (Darmstadt, Germany). The solvents used for liquid chromatography–mass spectrometry (LC–MS) analyses were of high-performance grade. Phytohormone standards, i.e., abscisic acid (ABA), indole-3-acetic acid (IAA), indole-3-butyric acid (IBA), indole-3-pyruvic acid (IPA), phenylacetic acid (PAA), brassinolide (BL), 28-homobrassinolide (HBL), 24-epibrassinolide (epiBL), 28-norbrassinolide (norBL), 24-epicastasterone (epiCS), cathasterone (CT), 6-deoxytyphasterol (6dTY), *cis*-zeatin (cZ), *cis*-zeatin-riboside (cZR), *cis*‐zeatin‐9‐glucoside (cZ9G), *cis*-zeatin-*O*-glucoside riboside (cZOGR), *trans*-zeatin (tZ), *trans*-zeatin-riboside (tZR), *trans*-zeatin-*O*-glucoside (tZOG), *trans*-zeatin-*O*-glucoside riboside (tZOGR), *trans*-zeatin-9-glucoside (tZ9G), *trans*-zeatin-9-glucoside-*O*-glucoside (tZ9GOG), dihydrozeatin (DHZ), dihydrozeatin riboside (DHZR), dihydrozeatin-*O*-glucoside (DHZOG), dihydrozeatin-*O*-glucoside riboside (DHZOGR), dihydrozeatin-7-glucoside (DHZ7G), dihydrozeatin-9-glucoside (DHZ9G), *N*^6^-isopentenyladenine (IP), *N*^6^-isopentenyladenosine-7-glucoside (IPR7G), *o*-topolin (oT), *m*-topolin (mT), *p*-topolin (pT), benzyladenine (BA), and gibberellic acid (GA_3_) were purchased from OlChemIm (Olomouc, Czech Republic).

### Phytohormone extraction

For the measurement of phytohormones, 500 mg of composts and 20 mg of biohumus (after evaporation) were placed into the 2 mL Eppendorf tubes, suspended in 1 mL 50% (*v/v*) acetonitrile (ACN) and homogenized in a bead mill homogenizer (3 cycles / 3 min, speed 3.10 m s^−1^; OMNI International a PerkinElmer company, Kennesaw, GA, USA) using two 3 mm tungsten balls. Then, samples were homogenized using the ultrasound processor VCX 130 (power 130 W, frequency 20 kHz, 5 min) equipped with a titanium probe (Sonics & Materials Inc., Newtown, CT, USA) and mixed in a laboratory shaker (90 rpm, dark, 5 °C, 30 min; LC-350, Pol-Eko-Aparatura, Poland). Samples were centrifuged (9000 × g, 5 min; MPW-55, Med. Instruments, Gliwice, Poland) and collected in a glass tube. For quantification of phytohormones, stable isotope-labeled standards of [^2^H_6_] ( +)-*cis*, *trans*-ABA (50 ng), [^2^H_5_] IAA (30 ng), [^2^H_6_] IP (50 ng), [^2^H_5_] tZ (30 ng), [^2^H_5_] tZOG (30 ng), [^2^H_3_] DHZR (30 ng), [^2^H_2_] GA_3_ (30 ng), [^2^H_3_] BL (20 ng) and [^2^H_3_] CS (20 ng) were added to samples as internal standards. Prepared extracts were purged using Waters SPE Oasis® HLB cartridge (Waters Corporation, Milford, MA, USA), previously activated and equilibrated using 1 mL 100% methanol (MeOH), 1 mL H_2_O, and 1 mL 50% (*v/v*) ACN. Then, extracts were loaded and collected to the Eppendorf tubes and eluted with 1 mL 30% (*v/v*) ACN. Samples were evaporated to dryness by centrifugal vacuum concentrator (Labconco CentriVap micro IR; Labconco Corp. Kansas City, MO, USA), dissolved in 50 μL 30% (*v/v*) ACN, and transferred into the insert vials.

### LC–MS analysis of phytohormones

Targeted compounds were analyzed using a LC–MS 8050 system consisting of a pump, degasser, autosampler, column oven, and mass spectrometer with triple quadrupole (Shimadzu Corporation, Kyoto, Japan). 10 μL of each sample was injected into the Waters XSelect C_18_ column (250 mm × 3.0 mm, 5 μm) (Waters Corporation, Milford, MA, USA), heated up to 50 °C. Mobile phase A was 0.01% (*v/v*) formic acid (FA) in ACN and phase B 0.01% (*v/v*) FA in H_2_O; the flow was 0.5 mL min^−1^. Separation of the above hormones was done in ESI positive mode with the following gradient: 0–8 min flowing increased linearly from 5 to 30% A, 8–25 min 80% A, 25–28 min 100% A, 28–30 min 5% A. The mobile LC phase consisted of binary gradients of ACN with 0.01% (*v/v*) formic acid (FA) (A) and 0.01% (*v/v*) aqueous FA (B), flowing at 0.5 mL min^−1^, which depended on the ESI mode, as described below. Analytical data were analyzed using Shimadzu BrowserWorkstation Software for LC–MS (Shimadzu Corporation, Kyoto, Japan).

### Statistical analysis

All the results are presented as mean values ± standard deviation (SD) of four biological replicates. Before selecting the appropriate statistical analysis method, the data were tested for normality (Shapiro–Wilk test) and homogeneity of variances (Levene’s test). The normality of data and homogeneity of variances were reported. Therefore, the data were analyzed using one-way ANOVA and the *F*-test established that there are statistically significant differences between calculated means. The means were grouped using Tukey’s post hoc test. The level of significance in all statistical tests was *p* ≤ 0.05. Hierarchical cluster analysis was applied to build a dendrogram which grouped data into a tree of clusters based on distances between all pairs of objects. A dendrogram of obtained clusters was created with Euclidean distance, while the agglomerative criterion was set to Ward’s method. After that, principal component analysis (PCA) was performed to build the relationship model between variables. The first twenty-three factors were preserved in a biplot for further analysis. The final biplot was created using two main components (PC1, PC2), which together explain 96.9% of the total variance. Statistical analyses were performed in Statistica 13.3 software (TIBCO Software Inc., Palo Alto, CA, USA)^37^.

## Data Availability

The datasets used and analysed during the current study are available from the corresponding author upon reasonable request.

## Abbreviations

ABA: Abscisic acid
BA: Benzyladenine
BE: Biohumus extract
BL: Brassinolide
CBH: Compost with buckwheat husk
CHCAP: Compost hemp chaff and apple pomace
CT: Cathasterone
cZ: *cis*-Zeatin
cZ9G: *cis*-Zeatin-9-glucoside
cZOGR: *cis*-Zeatin-*O*-glucoside riboside
cZR: *cis*-Zeatin-riboside
6dTY: 6-Deoxytyphasterol
DHZ: Dihydrozeatin
DHZ7G: Dihydrozeatin-7-glucoside
DHZ9G: Dihydrozeatin-9-glucoside
DHZOG: Dihydrozeatin-*O*-glucoside
DHZOGR: Dihydrozeatin-*O*-glucoside riboside
DHZR: Dihydrozeatin riboside
epiBL: 24-Epibrassinolide
epiCS: 24-Epicastasterone
GA_3_: Gibberellic acid
GC: Garden compost
HBL: 28-Homobrassinolide
IAA: Indole-3-acetic acid
IBA: Indole-3-butyric acid
IP: *N*^6^-Isopentenyladenine
IPA: Indole-3-pyruvic acid
IPR7G: *N*^6^-Isopentenyladenosine-7-glucoside
mT: *m*-Topolin
norBL: 28-Norbrassinolide
OC: Organic compost
OC-P: Organic compost-pellets
oT: *o*-Topolin
PAA: Phenylacetic acid
pT: *p*-Topolin
SA: Salicylic acid
tZ: *trans*-Zeatin
tZ9G: *trans*-Zeatin-9-glucoside
tZ9GOG: *trans*-Zeatin-9-glucoside-*O*-glucoside
tZOG: *trans*-Zeatin-*O*-glucoside
tZOGR: *trans*-Zeatin-*O*-glucoside riboside
tZR: *trans*-Zeatin-riboside
